# Efficiency and optimal size of hospitals: Results of a systematic search

**DOI:** 10.1371/journal.pone.0174533

**Published:** 2017-03-29

**Authors:** Monica Giancotti, Annamaria Guglielmo, Marianna Mauro

**Affiliations:** Department of Clinical and Experimental Medicine, Magna Graecia University, Catanzaro, Italy; University of South Australia, AUSTRALIA

## Abstract

**Background:**

National Health Systems managers have been subject in recent years to considerable pressure to increase concentration and allow mergers. This pressure has been justified by a belief that larger hospitals lead to lower average costs and better clinical outcomes through the exploitation of economies of scale. In this context, the opportunity to measure scale efficiency is crucial to address the question of optimal productive size and to manage a fair allocation of resources.

**Methods and findings:**

This paper analyses the stance of existing research on scale efficiency and optimal size of the hospital sector. We performed a systematic search of 45 past years (1969–2014) of research published in peer-reviewed scientific journals recorded by the Social Sciences Citation Index concerning this topic. We classified articles by the journal’s category, research topic, hospital setting, method and primary data analysis technique. Results showed that most of the studies were focussed on the analysis of technical and scale efficiency or on input / output ratio using Data Envelopment Analysis. We also find increasing interest concerning the effect of possible changes in hospital size on quality of care.

**Conclusions:**

Studies analysed in this review showed that economies of scale are present for merging hospitals. Results supported the current policy of expanding larger hospitals and restructuring/closing smaller hospitals. In terms of beds, studies reported consistent evidence of economies of scale for hospitals with 200–300 beds. Diseconomies of scale can be expected to occur below 200 beds and above 600 beds.

## Introduction

In many countries, the hospital sector has been involved in a massive reform process marked by financial restructuring of existing hospitals, mergers and closures of several small hospitals.

Healthcare organizations are required to achieve efficiency and effectiveness; they must reduce costs and offer quality health services [1]. One important source of potential inefficiency in the hospital sector relates to hospitals’ scale and scope. It might make good economic sense to enlarge the size and scope of a hospital to make better use of available expertise, infrastructure and equipment. However, at some point, a hospital departs from its optimal level of efficiency and begins to exhibit diseconomies of scale. At the other end of the scale, small hospitals might also be inefficient because the fixed infrastructural and administrative costs are shared across too small a caseload, thereby pushing up the cost of an average hospital visit.

In this context, the ability to measure scale efficiency is crucial to address the question of optimal productive size and to manage a fair allocation of resources. Most recent studies on scale efficiency in the healthcare sector focussed on analysing the proper use of resources [2, 3] and on estimating the optimal size of a hospital to increase the hospital’s performance [4, 5].

The question concerning scale efficiency is whether larger hospitals are more or less efficient than smaller ones. Research undertaken largely in the USA and the United Kingdom indicates that diseconomies of scale can be expected to occur below approximately 200 beds and above 600 beds [6]. Scale efficiency indicates the ability of a decision-making unit (DMU) to identify the "good" productive size in terms of resources used–*optimal size–*that allow the DMU to take full advantage of economies of scale by producing maximum output per unit of input and reducing the average unit costs of production.

According to the illustration of economies of scale, increasing the size of a very small operating unit (assigning, for example, double or triple resources) allows realizing economies of scale, i.e., the product increases more than twice or more than three times. Thus, the existence of economies of scale implies that there could be efficiency gains available by expanding firm size.

The optimum size is, therefore, that seen when all economies of scale have already been exploited but have not yet presented diseconomies. This consideration justifies the keywords for our search.

The paper is aimed at expanding and upgrading previous reviews on the optimal size of hospitals and contributing to the performance-management research literature as follows. There has been a particularly large increase in research on the topic in recent years due to the massive process of health integration and to the interest in finding an optimal size for hospitals as a reply to worldwide policies of financial constraint, which has led to greater public attention to the use of public resources. The number and wide range of publications justifies a review that allows, on the one hand, systematization of the literature and, on the other hand, identification of areas not treated, whose study will contribute to the evolution of science in terms that relate not only to knowledge but also to proactivity. We tabulated, reviewed, and synthesized studies related to scale efficiency published in the period 1969–2014. Our objectives were to analyse most topics investigated, the authors’ conclusions in this field and the methods used for the analysis and measurement of efficiency in the hospital sector. Moreover, the review allowed the identification of gaps in published research, suggesting opportunities for future research. The study will provide future scale-efficiency researchers with much to consider, leading to a “new” knowledge base for the scale-efficiency research field. The paper is organized as follows. In the next section, a description of the search strategy is provided. Then, we present our results. Finally, our conclusions are provided.

## Materials and methods

This systematic search was conducted according to the Preferred Reporting Items for Systematic Reviews and Meta-analyses (PRISMA) statement [7] (S1 Table).

### Search strategy

Our investigation begins with the definition of the problem about which we want to investigate.

We delineated the problem through 4 search questions: 1) Have mergers contributed to enhance hospitals efficiency? 2) Which is the optimal size of hospitals in terms of beds? 3) Which factors influenced the hospitals scale efficiency? 4) Finally, which are the most methods used in literature to analyse the hospitals scale efficiency?

Having delineated well-defined questions, we created a search strategy.

The identification of the search strategy has required the definition of a series of aspects concerning the findings process, as illustrated below.

First, the temporal extension of analysis. Because this attempt is the first to make a systematic search concerning efficiency and optimal size in the hospital sector, we started from the last review on this topic, specifically, the article by Hefty (1969), *“Returns to scale in hospitals*: *a critical review of recent research”* [8]. The author presented one survey of empirical studies concerning economies of scale and hospital costs from 1952 to 1969, finding that the long-term average cost curve appears to be U-shaped, with minimum average costs at the level of 200–300 beds. Accordingly, we collected articles from 1969 to 2014. Second, we determined the choice of the database from which to find papers. We chose the SSCI database (Social Science Citation Index), incorporated in the Web of Science Internet library source. We extracted papers from the SSCI database using 5separate keyword pairs (*scale efficiency*, *scale economies*, *hospital beds*, *hospital mergers*, *and hospital size*) to find the most articles focussed on this topic. In addition, we used 2 keywords focusing on the healthcare setting (*Hospital*, *Healthcare organization*), using the Boolean operator AND to identify all relevant papers in the field and to classify articles according to the covered issue. The search on the database by selected keywords has been extended to title, keywords and abstracts (topics range). The initial records found numbered 22.841articles. To overcome limits related to the choice and use of a single database, we integrated papers on the topic by using the Google search engine; the search returned 2.070.501 papers. The total of initial records was 2.093.342 papers. This large number is not surprising given the general nature of the search. Regardless, it is common in review articles to find a large number of records in early rounds of searching.

We browsed those publications, removed duplicates, determined their relevance and then further downloaded those that were relevant. In order to reduce the selection bias three members of the research team reviewed the titles, abstracts, and keywords of all records, which were retrieved separately, to determine whether the studies met the inclusion criteria. If there was any disagreement, it was resolved by discussion [9]. Our analysis is necessarily limited to publicly available papers and thus potentially subject to publication bias.

The initial results revealed several articles without direct connection to the precise review requirements because the review located all articles that contained the words “*scale efficiency”*, *“hospital beds”*, *“mergers”*, *“economies of scale”* or “*hospital size”*. Therefore, another round of searching was performed on these articles using the same terms in the search bar in the PDF version of the individual article. The results were then physically examined to determine the extent to which they carried insights and experiences related to scale efficiency.

Based on these rules, in the first phase of the selection process, we selected 131 papers and excluded 2.093.211 papers. All included studies were reviewed independently and in duplicate. In the second phase, of the 131 articles, we included only those journals with the Thomson Reuters Impact Factor published in 2013, as a proxy for the influence of publications. According to this rule, we eliminated 26 papers.

**Fig 1:**
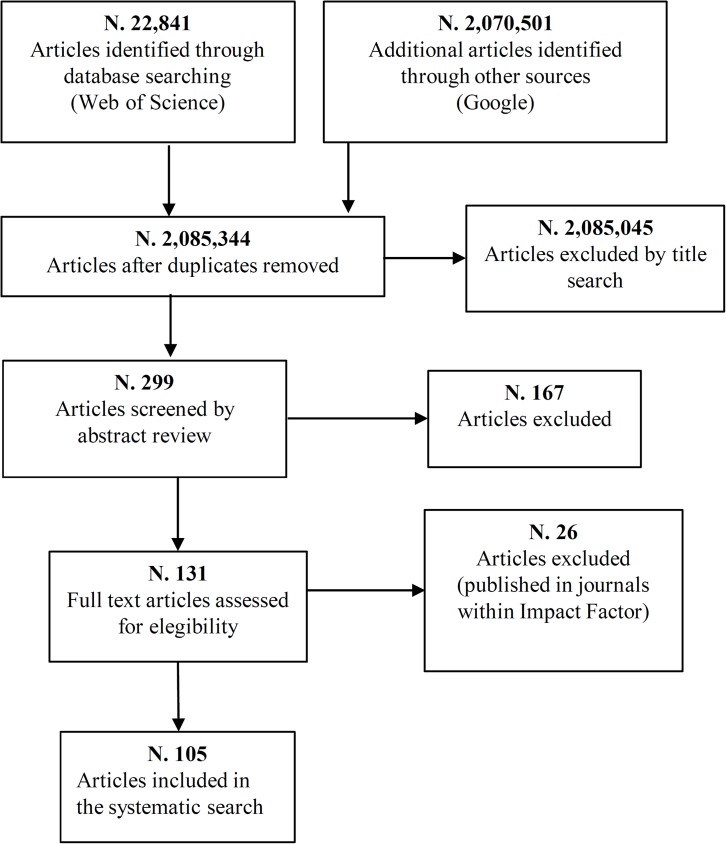
Flow diagram of the selection of articles included in the systematic search. Summary of study selection process. N.: number. doi:10.1371/journal.pmed1000097.

The final list consisted of 105 articles published in 43 journals. Fig 1 shows the flow diagram of the selection of articles included in the systematic search.

After the selection of papers, we classified journals in 4 macro subject areas according to the category/classification proposed by the Journal Citation Reports 2013, Thomson Reuters. For articles not included in Web of Science, we used the classification proposed by SJR (Scimago Journal & Country Rank), a portal that includes the journals and country scientific indicators developed from the information contained in the Scopus database (Elselvier B.V.).

Articles were classified as follows: 25 articles published by 13 *Business and Economic* journals; 45 articles published by 14 *Health Care Science and Services* journals; 12 articles published by 10 *Medicine* journals and 23 articles published by 6 *Operations Research and Management Science* journals (See S1 Appendix for the journals’ list). Ultimately, we reviewed 105 articles.

Following the framework of Shields [10]–also used by Hoque [11], Chenhall and Smith [12] and Hesford et al. [13]–the published articles were classified by (a) topic, (b) hospital setting, (c) research method, and (d) primary data analysis technique.

The identification of the different topics, research setting, research method and primary data analysis was informed by the literature of the last 45 years. Data synthesis involved a descriptive summary of included studies, as in the following sections.

### Classification of articles by topic

Analysis of all of the articles identified three macro categories within the topics investigated.

Decades of research were classified as follows:

1. Hospital cost Efficiency, or analysis of potential cost gains arising from hospital mergers. Most of these studies were concentrated in the period 1969–1989. Specifically, this macro category included the following topics:
  - *Hospital cost efficiency*. Articles in this area discussed hospital cost characteristics of hospitals in relation to size (large and small hospitals). Authors explored hospital costs through a comparison of different hospitals in terms of size and activity.
  - *Hospital mergers and cost saving*. Authors investigated the effect of mergers on hospital costs. In general, results supported the policy of expanding larger hospitals and restructuring/closing smaller hospitals. However, results also indicate that above a certain size, the expansion of large hospitals might not yield substantial efficiency gains in terms of cost.
  - *Optimal size of hospitals*. Under increasing pressure to cut costs, hospitals have tried to increase their occupancy levels by significantly reducing the number of inpatient beds through downsizing, mergers and hospital closings. The question under analysis is, what is the optimal size of hospitals in terms of beds that allows the containment of costs but provides, at the same time, the maximum level of productive efficiency? Studies reported consistent evidence of economies of scale for hospitals with 200–300 beds. However, increasing return to scale are present until 600 beds [14].
2. Technical and Scale efficiencies of hospitals. Technical efficiency is a concept that aims to evaluate whether a productive unit is using the minimum possible quantity of resources. Evaluation is achieved through the relationship between input (e.g., hospital beds and hours of work by physicians) and output (e.g., number of ordinary admissions and medical outpatients). One productive unit can be far more efficient (in a technical sense) depending upon how much higher this relationship is. One inadequate technical efficiency score might depend upon an operational scale that is inappropriate (e.g., too large or too small). In this case, it is necessary to address the question of “economies/diseconomies of scale”. According to the illustration of economies of scale, increasing the size of a hospital unit can result in product increases of more than double or more than triple; therefore, unit production costs decrease. Most of the studies in this area were concentrated in the period 1990–2000. This macro category included the following topics:
  - *Frontier efficiency measurement*. Studies in this area investigated frontier efficiency measurement methods. Results showed that the number of studies that seek to measure health-service efficiency and productivity continues to increase quite dramatically. Concerning methods, the techniques used are largely based on nonparametric Data Envelopment Analysis (DEA).
  - *Technical and scale efficiency score*. Studies under this topic assigned a score in terms of technical or scale efficiency to a sample of hospitals.
  - *Scale efficiency of hospitals*. The main objective of this empirical study was to verify the existence of scale economies and to estimate the Most Productive Scale size in hospital samples using a parametric or non-parametric method such as DEA or Stochastic frontier analysis (SFA).
  - *Technical and scale efficiency effect on quality of care*. An important question was the relationship between technical and scale efficiency and quality of care [15]. Results showed that quality of care was significantly positively related to technical efficiency but significantly negatively related to scale efficiency.
  - *Sources of Inefficiency*. Authors analysed technical and scale efficiency of hospitals in a first phase. In a second phase, they investigated sources of inefficiency. Some articles showed that the size of hospitals is a major source of inefficiency [16].
  - *Effect of market and organizational structure on hospitals’ efficiency*. Papers investigated the efficiency of hospitals and showed that the differences in efficiency scores can be attributed to several environmental factors such as market structure and organizational structure.
3. Effect of healthcare reforms, managerial aspects and ownership on hospital efficiency. Studies in this area provided insights into how hospitals responded to the pressure for increased efficiency and quality introduced by the reforms. These topics are those most frequently seen in studies published from 2001 to 2014. Topics include the following:
  - *Efficiency effect of health reforms*. Technical and scale efficiencies of a sample of hospitals were comparatively examined before and after the reform to control health expenditures to observe the benefits from the reforms.
  - *Effect of ownership on hospital efficiency*. Studies in this area investigated the effect of ownership on hospital efficiency. Specifically, some studies compared efficiency of private, public, for profit and non-profit hospitals to analyse the differences. Empirical results indicated that private and non-profit hospitals are on average less cost efficient and less technically efficient than are publicly owned hospitals [17].

### Classification of articles by method

Concerning the research method applied, it is possible in our review to identify the following approaches:

- *Theoretical study*. Authors present a theoretical explanation of the different models used for the assessment of economies of scale (e.g., DEA and SFA) or discuss theoretically the question under analysis.
- *Descriptive study*. Authors describe the health care system under analysis or research setting and variables used in the analysis.
- *Empirical study*. Empirical research is a means of gaining knowledge by means of direct and indirect observation or experience. Empirical evidence can be analysed quantitatively or qualitatively. Through quantifying the evidence, a researcher can answer empirical questions, which should be clearly defined and answerable with the evidence collected (usually called data). Specifically, studies that used this method focussed on analysing the technical and scale efficiency of a sample of hospitals. The data presented reveal that empirical study methods were the most frequently used. The presence of a large number of empirical studies might be explained by the nature of the topic analysed in this paper.
- *Review*. This method includes a literature analysis.

Mixed approaches:

- *Theoretical/Descriptive study*;
- *Theoretical/Empirical study*;
- *Descriptive/Empirical study*;
- *Theoretical/Descriptive and Empirical study*.

### Classification of articles by research setting

Concerning research setting, we classified hospitals according to *services offered* (Hospital types), *location* (Urban and Rural Hospitals), and *ownership* (Public, Private and Church Hospitals).

Concerning *service offered*, hospitals in the sample were classified as follows:

- *General/Acute-care Hospitals*. These facilities are set up to address many types of disease and injury. Normally, there is an emergency department to address immediate and urgent threats to health.
- *District Hospitals*. A district hospital typically is the major health care facility in its region, with large numbers of beds for intensive care and long-term care.
- *HMOs*. A health maintenance organization (HMO) is an organization that provides or arranges managed care for health insurance, self-funded health care benefit plans, individuals, and other entities in the United States, and it acts as a liaison with health care providers (e.g., hospitals and doctors) on a prepaid basis. Unlike traditional indemnity insurance, an HMO covers care rendered by those doctors and other professionals who have agreed by contract to treat patients in accordance with the HMO's guidelines and restrictions in exchange for a steady stream of customers.
- *Teaching hospitals*. A teaching hospital combines assistance to people with teaching medical students and nurses and often is linked to a medical school, nursing school or university.
- *Hospital units*. In some cases, the analysis is performed within hospital units. Authors evaluated economies of scale and scope when comparing different hospital units or services.
- *Mixed sample*. Some studies used a mixed sample comprising teaching, general, and other hospital types. Authors found that economies of scale and scope depended upon the category of the hospital in addition to the number of beds and volume of output.
- *Non-specified*. These studies examine a sample of non-specified hospital types.

Concerning *hospital location*, authors’ choices were associated with analysis goals.

We could differentiate *rural hospitals* and *urban hospitals*. An urban hospital is generally located in a metropolitan area and serves a large range of people. Rural hospitals are much smaller, particularly in terms of beds. However, urban hospitals, despite the high demand for services, are very much better supplied with resources than are corresponding rural hospitals. Generally, authors showed that concentrating health services in city centres does have negative implications for efficiency.

Most authors included urban and rural Hospitals to compare hospitals in terms of size and location. We included these studies under the classification *All Types*.

Finally, we classified articles according to *hospital ownership* considered in the sample. Specifically, we distinguished among the following:

- *Government Hospitals (Public Hospital)*. The popular choice of settings for scale efficiency studies was public hospitals, which is not surprising, given that the process of restructuring and downsizing concerned primarily public hospitals. The greatest number of mergers focussed on the public hospital sector to contain health expenditures and to rationalize use of resources.
- *Non-Government Hospitals (Private Not-for-profit; Private For Profit)*. A non-profit hospital, or not-for-profit hospital, is a hospital that is organized as a non-profit corporation. For-profit hospitals are investor-owned chains of hospitals that were established particularly in the United States in the late twentieth century. In contrast to the traditional and more common non-profit hospitals, these chains attempt to garner a profit for their shareholders.
- *Mixed Sample*. Studies that used a sample composed of different hospitals, in terms of ownership, aimed to understanding whether there were differences between hospitals’ type in terms of technical and scale efficiency. In this context, many articles aimed to analyse whether public hospitals were more or less efficient than were private facilities. Most authors showed that public hospitals appeared more efficient than did private hospitals.
- *All Types*. This sample type included Government, Non-Government and Church Hospitals.
- *Non-specified*. These studies examined non-specified hospital types according to ownership.

### Classification of articles by primary data analysis technique

We classified articles according to quantitative and qualitative methods used to conduct their analyses.

Concerning *quantitative methods*, we distinguish among these different techniques:

- *DEA analysis*: The most frequently employed quantitative method is DEA, a non-parametric approach that estimates efficiency scores from sample data by using linear programming techniques. DEA remains the preferred method of efficiency analysis in the non-profit sector in which there is multiple-output production and it is difficult to obtain input and output price data or to make behavioural assumptions such as profit maximization or cost-minimization.
- *Stochastic Frontier Analysis*: SFA is an alternative method to examine hospital efficiency. The analysis of production and costs in the stochastic frontier framework involves two steps. First, the frontier model is estimated. Second, the estimated model is used to construct measures of inefficiency or efficiency.
- *Cost Function Model*: Studies in this area used a cost function model to express production costs through a cost function in terms of the amount produced.
- *Queueing Model*: Some articles used a queueing model to estimate the scale efficiency of hospitals. Queueing theory is the mathematical study of waiting lines, or queues. In queueing theory, a model is constructed so that queue lengths and waiting times can be predicted. Queueing theory is generally considered a branch of operations research because the results are often used when making business decisions concerning the resources needed to provide a service.
- *Cobb-Douglas Functional Form*: This mathematical function, formulated by Cobb and Douglas, is widely used in economic analyses. The function describes how the product varies in relation to changes in the factors of production (production function) or in the amount of other goods (utility function).
- *Mixed methods*: This section included articles that used mixed methods.
- *None*: For theoretical studies, quantitative methods correspond to none.

Concerning *qualitative methods*, authors collected data using the following sources:

- *Official database*: This database contains data from the Ministry of health, annual statistical publications, official discharge records and casemix data.
- *Direct Contact*: Some studies used direct contact, such telephonic interviews or questionnaires to collect data and information.
- *Mixed sources*: This section included studies that used mixed sources.
- *Non-specified*: This classification is being used in this paper to conduct the review.

## Results

### Temporal distribution of articles

Table 1 presents the temporal distribution of articles on scale efficiency of hospitals published by *Business* and *Economics*, *Health Care Sciences*, *Medicine* and *Operational research and Management Science* journals.

**Table 1:** Temporal distribution of articles by journals.

JOURNAL	1969–1989	1990–2000	2001–2014	TOTAL	TOTAL%
*Business and Economics Journals*	0	9	16	25	24%
*Health Care Science and Services Journals*	4	7	34	45	43%
*Medicine Journals*	1	3	8	12	11%
*Operational Research and Management Science Journals*	3	6	14	23	22%
**TOTAL**	*8*	*25*	*72*	*105*	*100%*

Table 1 shows an important increase in the number of publications on the topic. In the first decade of the study, authors concentrated on the shape of the hospital industry's cost function and on the importance of the relationship between hospital costs and the scale of output, called "returns to scale" or "economies of scale”.

The authors recognized the failure of smaller hospitals to produce all of their individual product lines in efficient volumes. The implication was that large hospitals might have a greater potential for scale economies. Thus, since the mid-1970s, successive governments in the UK and the USA have made continuous efforts to find ways of improving efficiency and curtailing expenditures in the National Health Service.

New approaches to public sector management were introduced beginning in 1980.

One strategy for cost containment was the concentration of hospital services in large hospitals, with the associated closure of a number of smaller hospitals.

Authors’ interest increased beginning in 1990, in accordance with the wave of vertical integrations and horizontal mergers that occurred in the United Kingdom and the USA. In the USA, the early 1990s could also be labelled a restructuring era for health care systems. One important reason for vertical integration was lowering transaction costs, increasing production cost savings, responding to management and internal factors, and environmental changes. Conversely, the primary motives behind horizontal mergers were potential economies of scale and increasing market power.

From 1990 to 2000, publications concerning this topic increased. Authors discussed the potential implications of the restructuring of the health care industry for competition, efficiency, and public policy.

Starting in 2000, hospital industries worldwide have indeed been focussed on a huge process of reorganization; we found 72 articles published from 2001 to 2014. To contain the presence of excess capacity, public producers' numbers of beds have been reduced by Central or Regional governments almost everywhere.

Additionally, a number of mergers continued to grow to exploit economies of scale and scope and to improve the effectiveness and quality of care.

### Studies published in Business and Economic journals

In this section, we examine 25 articles published in 13 Business and Economics journals that address the scale efficiency of public hospitals.

### Frequency distribution of articles by Business and Economics journals

Table 2 presents the frequency distribution of articles on scale efficiency of hospitals published by 13 Business and Economics journals. Most of the articles were published by JPA or AE. Specifically, JPA was an appropriate journal in this field, publishing theoretical and applied research addressing the measurement, analysis, and improvement of productivity. The measurement of productivity in this context is an important question to understand whether one hospital is efficient, i.e., whether resources are properly used.

**Table 2:** Frequency distribution of articles published by Business and Economics journals.

**JOURNAL**	**1969–1989**	**1990–2000**	**2001–2014**	**TOTAL**	**TOTAL %**
JPA	0	1	5	6	23%
AE	0	5	1	6	23%
RSUE	0	0	2	2	8%
SAJEMS	0	0	2	2	8%
CER	0	0	1	1	4%
EI	0	0	1	1	4%
EK	0	0	1	1	4%
EM	0	0	1	1	4%
ESR	0	1	0	1	4%
JAE	0	0	1	1	4%
JEP	0	1	0	1	4%
RIO	0	0	1	1	4%
RvE&S	0	1	0	1	4%
**TOTAL**	*0*	*9*	*16*	*25*	*100%*

JPA: Journal of Productivity Analysis; AE: Applied Economics; RSUE: Regional Science and Urban Economics; SAJEMS: South African Journal of Economics; CER: China Economic Review; EI: Economic Inquiry; EK: Ekonomicky Casopis; EM: Economic Modelling; ESR: Economic and Social Review; JAE: Journal of Applied Econometrics; JEP: Journal of Economic Perspectives; RIO: Review of Industrial Organization; RvE&S: The Review of Economics and Statistic.

### Research topics

Table 3 shows the frequency distribution of scale efficiency topics for the 25 articles published in the period under study.

**Table 3:** Frequency distribution of articles published in Business & Economic journals by research topic.

TOPIC	1969–1989	1990–2000	2001–2014	TOTAL		TOTAL%
*Hospital cost efficiency*						
Hospital cost efficiency	0	2	3	5	20%	
Hospital mergers and cost saving	0	4	3	7	28%	
Optimal size of hospitals	0	0	1	1	4%	
*Technical and Scale Efficiencies of Hospitals*						
Frontier Efficiency Measurement	0	0	0	0	0%	
Technical and Scale efficiencies score	0	0	1	1	4%	
Scale efficiency of hospitals	0	3	2	5	20%	
Sources of Inefficiency	0	0	1	1	4%	
Technical and Scale efficiencies effect on Quality of care	0	0	2	2	8%	
Effect of market and organizational structure on hospitals’ efficiency	0	1	0	1	4%	
*Effects of Healthcare Reforms*, *Managerial Aspects and Ownership on Hospital Efficiency*						
Efficiency effect of health reforms	0	0	1	1	4%	
Effect of ownership on hospital efficiency	0	0	1	1	4%	
**TOTAL**	*0*	*10*	*15*	*25*	*100%*	

Generally, *Business and Economic* journals welcome articles in all areas of business and economics research. Accordingly, we found articles that discussed different research topics.

Twenty per cent of the articles (5) were focussed on analysis of *hospital cost efficiency*.

Authors found that hospitals’ cost inefficiency was often due to using too many input resources (number of personnel and beds–technical inefficiency), although the use of a wrong mix of resources (allocative inefficiency) also raised costs [18]. Operating at non-optimal scale also raised hospitals' costs but to a lesser extent.

Authors concluded that hospitals could substantially reduce costs by adjusting their level and mix of input usage, thus reducing costs without sacrificing access. For example, occupancy rate of beds is a significant variable explaining the level of hospital costs [19]. When the existing number of beds is in excess of that required for efficient inpatient service provision, total beds could be reduced, thus producing the same output at lower cost [20].

However, comparing the efficiency and costs of different sets of hospitals operating in different institutional and competitive environments, authors found that hospitals operating in an environment that is heavily regulated perform better than did hospitals operating in one that is private and largely regulated [21].

Finally, Kibambe and Koch [22] found that services provided by small-scale medical facilities waste fewer resources and are more efficient in terms of costs compared with medical centres that offering more technical services.

Most articles (28%) were focussed on the question of *hospital mergers and related cost saving*.

In general, authors showed that efficiency gains could result from downsizing large hospitals [23]. The policy of expanding larger hospitals and restructuring/closing smaller hospitals was supported [24], but results also indicated that the expansion of large hospitals might not yield substantial efficiency gains in terms of productive efficiency [25].

In other studies, authors discussed the potential implications of the health care industry’s restructuring for competition, efficiency, and public policy or analysed potential cost gains resulting from mergers [26]. Generally, findings showed that economies of scale are present for merging hospitals and that they realize cost savings immediately following a merger. However, results showed that over time, cost savings from the merger decrease and the proportion of hospitals experiencing positive cost savings declines [27]. Finally, Cohen and Morrison Paul [28] evaluated scale, scope and agglomeration economies for Washington State hospitals from 1997 to 2002. For their primary focus, agglomeration economies, they found evidence that knowledge sharing through proximity to other hospitals was a significant cost-saving approach.

One article [29] investigated the *optimal size of hospitals*, specifically, the relationship between efficiency and the size or scale of the hospitals, using DEA.

The authors showed that different hospitals might have different optimal sizes or different efficient modes of operation, depending upon location, the population they serve, and the policies their respective provincial governments wished to implement.

One article analysed the efficiency of a sample composed of large and small hospitals and assigned a score in terms of *technical and scale efficiency*. According to the results, the authors found that small hospitals tend to be more efficient, whereas large hospitals tend to be less efficient [30].

Five articles in Business and Economic journals focused on the evaluation of *scale efficiency in hospitals*. The main objective of this empirical study was to verify the existence of scale economies in hospital samples using parametric or non-parametric methods such as DEA, SFA, or through a cost function [31, 32]. Generally, studies showed increasing returns to scale among hospitals above the median size (more than 300 beds) and until 600 beds [14]. Finally, Goncalves and Barros [33] evaluated economies of scale in the provision of services within Portuguese hospitals, particularly auxiliary clinical services. Such services have a significant weight in total hospital costs; a proper analysis of their cost structure is important. The authors found the existence of economies of scale and scope.

An important question, explored only in one study, concerned the *relationship between technical and scale efficiency and quality of care*. In general, results showed that quality of care was significantly positively related to technical efficiency but significantly negatively related to scale efficiency [15]. Moreover, Bilodeau et al. [34] suggested that observable differences in management methods or unmeasurable differences in the quality of care underlie the differences in observed performance.

One study investigated *sources of inefficiency* in hospitals. The author demonstrated that, in some cases, pure technical inefficiency was the driving force for pulling down the overall efficiency of these hospitals [35]. Hospitals’ inefficiency reflected the revenue-based behaviour of hospitals in which unnecessary care, over-prescription of drugs, and the adoption of high-tech treatments were commonly found.

The *Effect of market structure on technical and scale efficiency* was analysed in one study [36]. The paper investigated the effect of market structure on the technical efficiency of hospitals, decomposed into pure technical and scale efficiency. The authors showed that differences in efficiency scores are attributable to several environmental factors, such as market structure and regulation effects. Specifically, DEA results showed a positive influence of market structure on the level of efficiency. The presence of competitors in the local market, independently of their market share, seemed to improve technical efficiency. The authors also found that hospital mergers justified by expected improvements in scale efficiency might have a negative counterpart to technical efficiency by eliminating potential competitors.

One study investigated *efficiency effect of health reforms*. Technical and scale efficiencies of a sample of Italian hospitals were comparatively examined before and after a reform to control health expenditures [37].

Findings suggested that a restructuring policy of the hospital industry involving only a reduction of beds was not a viable strategy for controlling public health care expenditures.

Given this evidence, one can notice that placing restrictions on bed capacity–without considering their limited possibility of substitution–might imply an inefficient use of resources and severely limit the possibility of controlling public health expenditures by restructuring the hospital industry.

Finally, Daidone and D’amico [38] investigated *Effect of ownership on hospital efficiency*, finding that inefficiency is negatively associated with specialization and positively associated with capitalization.

Capitalization is typical of private structures that, on average, use resources less efficiently compared with public and not-for-profit hospitals.

### Research topic in a hospital setting

Table 4 shows the frequency distribution of articles on efficiency published in *Business and Economic journals* by research topic in a hospital setting.

**Table 4:** Frequency distribution of articles published in Business & Economic journals by research setting.

RESEARCH SETTING					
**ACCORDING TO SERVICE**	**1969–1989**	**1990–2000**	**2001–2014**	**TOTAL**	**TOTAL%**
General/Acute-care hospitals	0	7	12	19	77%
District hospitals	0	0	0	0	0%
HMOs	0	0	0	0	0%
Hospital units	0	1	2	3	12%
Teaching Hospitals	0	0	0	0	0%
Mixed sample	0	1	0	1	4%
Non-specified	0	1	1	2	8%
**TOTAL**	*0*	*10*	*15*	*25*	*100%*
**ACCORDING TO LOCATION**	**1969–1989**	**1990–2000**	**2001–2014**	**TOTAL**	**TOTAL%**
Urban Hospitals	0	0	3	3	12%
Rural Hospitals	0	1	0	1	4%
All types	0	7	4	11	46%
Non-Specified	0	2	8	10	38%
**TOTAL**	*0*	*10*	*15*	*25*	*100%*
**ACCORDING TO OWNERSHIP**	**1969–1989**	**1990–2000**	**2001–2014**	**TOTAL**	**TOTAL%**
*Government Hospitals*					
Public Hospitals	0	2	10	12	50%
*Non-Government Hospitals*					
Private Not-For-Profit	0	0	0	0	0%
Private For Profit	0	0	0	0	0%
*Mixed sample*	0	6	4	10	38%
*All Types*	0	0	0	0	0%
*Non-specified*	0	2	1	3	12%
**TOTAL**	*0*	*10*	*15*	*25*	*100%*

HMO: Health Maintenance Organization.

Concerning services offered, the popular choice of setting for scale efficiency studies was *General/Acute-care hospitals* (77%). Studies in this setting increased from 2000.

In other cases, the analysis of hospital performance was performed within *hospital units* (12%). In this context, authors evaluated economies of scale and scope comparing different hospital units or services.

This choice was appropriate, particularly when the analysis compared homogeneous units.

One study used a *mixed sample* (4%) composed of teaching, base, small general, country, maternity, geriatric, psychiatric and maternity hospitals [23].

In two studies, *non-specified* hospital types constituted the sample (8%).

Concerning *hospital location*, choices were associated with analysis goals.

Most authors (46%) included *Urban* and *Rural Hospitals* to compare hospitals in terms of size.

Finally, concerning *hospital ownership*, the popular choice of setting for scale efficiency studies was *Public Hospitals* (50%). This choice is not surprising, given that the process of restructuring and downsizing concerned primarily public hospitals. The largest number of mergers concerned the public hospital sector to contain health expenditures and to rationalize use of resources. Studies that used a sample composed of different hospitals, in terms of ownership, aimed to understanding whether public hospitals are more or less efficient than private hospitals. Generally, public hospitals appeared more efficient than private hospitals. Nevertheless, 10 studies used a *mixed sample* composed of public and private hospitals, and 3 studies used non-specified hospital types, according to ownership.

### Research methods

Table 5 shows the frequency distribution of articles published in Business & Economic journals by research methods. The data presented reveal that *empirical studies* were the most frequently used, which was the case in14 articles (54%). The presence of a large number of empirical studies might be explained by the nature of the topic analysed in this paper. We found only one *theoretical study*. Specifically, Gaynor and Wilson [26] discussed the question of hospital mergers and potential implications of the restructuring of the health care industry for competition, efficiency, and public policy.

**Table 5:** Frequency distribution of articles published in Business & Economic journals by research method.

RESEARCH METHOD	1969–1989	1990–2000	2001–2014	TOTAL	TOTAL%
Empirical Study	0	4	9	13	54%
Descriptive Study	0	0	0	0	0%
Theoretical Study	0	1	0	1	4%
Review	0	0	0	0	0%
Mixed Methods	0	5	6	11	42%
**TOTAL**	*0*	*10*	*15*	*25*	*100%*

Eleven articles used *mixed methods* (42%). Specifically, we found 5 *theoretical/empirical studies*. The authors presented a theoretical explanation of the different models used for the assessment of economies of scale (e.g., DEA and SFA), or discussed theoretically the question under analysis. In a second phase, they applied the method of analysis to a sample of hospitals.

Four studies were *descriptive/empirical studies*. In these cases, before the empirical analysis, authors described the context of analysis (health reforms, regions or model). Finally, 2 studies were *theoretical/descriptive* and *empirical studies*. These papers were divided as follows. In a first stage, the authors discussed theoretically the question under analysis (hospital mergers, potential gains from the mergers and other topics). In a second stage, the authors described the model presented to calculate technical and scale efficiency of hospitals; finally, the model was applied to a sample of hospitals.

### Primary data analysis techniques

Table 6 shows the frequency distribution of articles published in Business and Economic journals by the primary data analysis technique. One study was a theoretical study [26]. For this reason, quantitative methods used corresponds to *none*. Concerning *quantitative methods*, the most frequently used analysis technique is *DEA* (50%). DEA remains the preferred method of efficiency analysis in the non-profit sector; in this method, there is multiple-output production and it is difficult to obtain input and output price data or to set behavioural assumptions such as profit maximization or cost-minimization.

**Table 6:** Frequency distribution of articles published in Business & Economic journals by PDAT.

PDAT					
**QUANTITATIVE**	**1969–1989**	**1990–2000**	**2001–2014**	**TOTAL**	**TOTAL%**
DEA Analysis	0	4	9	13	50%
Stochastic Frontier Analysis	0	0	2	2	8%
Cost Function Model	0	2	3	5	23%
Queueing Analysis	0	0	0	0	0%
Cobb-Douglas Functional Form	0	0	0	0	0%
Mixed methods	0	3	1	4	15%
None	0	1	0	1	4%
**TOTAL**	*0*	*10*	*15*	*25*	*100%*
**QUALITATIVE**	**1969–1989**	**1990–2000**	**2001–2014**	**TOTAL**	**TOTAL%**
Official database	0	9	14	23	92%
Direct contact	0	0	0	0	0%
Mixed sources	0	0	1	1	4%
Non-specified	0	1	0	1	4%
**TOTAL**	*0*	*10*	*15*	*25*	*100%*

PDAT: Primary Data Analysis Techniques.

Four studies employed *Mixed methods* (15%). Specifically, Frech and Mobley [31] tested scale efficiency in hospitals using parametric and non-parametric methods (Stigler's multivariate survivor analysis and probit model).

McCallion et al. [25] used Fare et al. distance function approach [39] and Malmquist Index to compare productive efficiency of a sample of larger and smaller hospitals. Daidone and D’amico [38] analysed the effect of productive structure and level of specialization of hospital on technical efficiency using the Cobb-Douglas function and the Stochastic Frontier Model. Finally, Ferrier and Valdmanis [18] used nonparametric, non-stochastic cost and production frontiers. Additionally, the study used Tobit Analysis to estimate cost, technical, allocative and scale efficiencies of public and private hospitals. Mixed methods was followed by *SFA* (8%).

Concerning *qualitative methods*, most of the studies make use of *official records* (92%), that is data from the Ministry of health, annual statistical publications, official discharge records and casemix data.

One study [22] used *mixed methods* (4%).

Specifically, data were collected in a first stage by official records. Those lacking data were collected through direct contact with hospitals. One study did not specify a data source (4%).

### Studies published in Health Care Science and Services journals

In this section, we examine 45 articles published by 14 *Health Care Science* and *Services* journals that address the scale efficiency of public hospitals.

### Frequency distribution of articles by Health Care Science and Services journal

Table 7 lists the number of articles on efficiency in the hospital sector published in 14 Health Care Science and Services journals during the period 1969–2014. We reviewed 45 articles. We observed increasing interest from the first to the last decade.

**Table 7:** Frequency distribution of articles published by Health Care Sciences and Services journal.

JOURNAL	1969–1989	1990–2000	2001–2014	TOTAL	TOTAL%
*HE*	0	3	6	9	20%
*HP*	0	0	6	6	13%
*JMS*	0	0	6	6	13%
*HSR*	3	1	1	5	11%
*JHE*	1	2	1	4	9%
*BMC HSR*	0	0	3	3	7%
*EJHE*	0	0	3	3	7%
*HCMR*	0	0	2	2	5%
*GHA*	0	0	1	1	2%
*HEPL*	0	0	1	1	2%
*HPMJ*	0	0	1	1	2%
*INQ*	0	0	2	2	5%
*JHCPU*	0	0	1	1	2%
*JRH*	0	1	0	1	2%
**TOTAL**	*4*	*7*	*34*	*45*	*100%*

HE: Health Economics; HP: Health Policy; JMS: Journal of Medical Systems; HSR: Health Services Research; JHE: Journal of Health Economics; BMC HSR: BMC Health Services Research; EJHE: European Journal of Health Economics; HCMR: Health Care Management Review; GHA: Global Health Action; HEPL: Health Economics Policy and Law; HPMJ: The International Journal of Health Planning and Management; INQ: INQUIRY: The Journal of Health Care Organization, Provision, and Financing; JHCPU: Journal of Health Care for the Poor and Underserved; JRH: Journal of Rural Health.

### Research topics

Generally, studies in *Health Care Science & Services journals* investigate how social factors, financing systems, organizational structures and processes, medical technology, and personal behaviours affect access to health care, quality of health care and cost of health care. Accordingly, we found in this section many articles that investigated the effect of reforms or of organizational structure on hospitals’ efficiency. All topics investigated are explained in Table 8. Four articles were found under the topic Hospital *cost efficiency*. Some authors [40, 41] investigated the development of hospital cost efficiency and productivity.

**Table 8:** Frequency distribution of articles published in Health Care Science and Services journals by research topic.

TOPIC	1969–1989	1990–2000	2001–2014	TOTAL	TOTAL%
*Hospital cost efficiency*					
Hospital cost efficiency	0	2	2	4	9%
Hospital mergers and cost saving	0	2	2	4	9%
Optimal size of hospitals	2	0	3	5	11%
*Technical and Scale Efficiencies of Hospitals*					
Frontier Efficiency Measurement	0	0	1	1	2%
Technical and Scale efficiencies score	0	1	8	9	20%
Scale efficiency of hospitals	2	2	3	7	16%
Sources of Inefficiency	0	0	2	2	4%
Technical and Scale efficiencies effect on Quality of care	0	0	2	2	4%
Effect of market and organizational structure on hospitals’ efficiency	0	0	2	2	4%
*Effect of Healthcare Reforms*, *Managerial Aspects and Ownership on Hospital Efficiency*					
Efficiency effect of health reforms	0	0	6	6	14%
Effect of ownership on hospital efficiency	0	0	3	3	7%
**TOTAL**	*4*	*7*	*34*	*45*	*100%*

Additionally, in this context, empirical results indicated that private and non-profit hospitals were on average less cost efficient and less technically efficient than were publicly owned hospitals [17]. Another relevant contribution was provided by Farsi and Filippini [42]. The authors explored the cost structure of Swiss hospitals, focusing on differences due to teaching activities and differences related to ownership and subsidization types. Results showed that teaching activities were an important cost-driving factor and that hospitals that had a broader range of specialization were relatively more costly.

Four articles investigated *Hospital mergers and cost saving*.

In general, articles in this area investigated the effects of mergers, analysing hospitals’ costs prior and after mergers [43].

Exploring empirically the effects of mergers in three areas (scale of operation, operating efficiency, and staffing practices), Alexander et al. [44] showed that the short-term effect of a merger was generally modest but differed according to the conditions under which the merger occurred. Specifically, mergers occurring later in the study period and mergers between similarly sized hospitals displayed greater change in operating characteristics than did mergers occurring earlier in the study period and mergers between hospitals of dissimilar size. Such differences are attribute to increased competitive pressures and to greater opportunities for consolidation and efficiencies in mergers involving similarly sized hospitals.

Investigating the operating efficiencies of merged and control hospitals prior to and after the merger, some authors [45] showed that hospital merger activity reduced the cost of production by achieving scale and scope economies, allowing hospitals to become more efficient. Moreover, in some cases, a moderate increase in quality seemed to stem from hospital mergers. In contrast, it seemed more rewarding to promote cross-functional collaboration together with clinical specialization [46].

Five articles discussed *optimal size of hospitals*. Analysing hospitals’ costs in relation to size [47, 48], some of articles found that economies of scale were present with an optimum hospital size of approximately 230 beds [4]. However, most authors found that large hospitals (over 300 beds) might have a greater potential for scale economies until 600 beds [49, 50].

We found a review of papers published on *Frontier efficiency measurement* methods [51]. The author reviewed 317 published papers on frontier efficiency measurement published from 1983 to 2006. Results showed that the techniques used were primarily based on nonparametric data envelopment analysis, but there was increasing use of parametric techniques such as stochastic frontier analysis.

Most articles in health care science and services journals (9) focused on the *Technical and scale efficiency* of hospitals.

Authors analysed technical and scale efficiency scores within different hospital samples [52, 53]. Results varied across countries. For example, Suraratdecha and Okunade [54] investigated the economic relationship among medical resources and efficiency of the health care system in a developing Asian country. Results showed that different types of medical care workers (doctors, nurses, and pharmacists) influenced efficiency differently. The marginal products of nurses and capital were the highest, and they varied across the regions.

Kirigia et al. [2] assessed technical and scale efficiency and productivity changes of public municipal hospitals in Angola. Results showed that, on average, productivity of municipal hospitals in Angola increased by 4.5% over the period 2000–2002; the growth was due to improvements in efficiency rather than innovation. Chilean primary healthcare practice was analysed using a DEA analysis multiple stage approach by Ràmirez et al. [53]. Results showed that urban hospitals were more efficient than rural hospitals. In addition, Ketabi [55] evaluated the performance of 23 Cardiac Care Units of hospitals in Isfahan, Iran. The author reveals that 11 of 23 units were inefficient. The results suggested improvement strategies based on output factors. Flokou et al.[56]analysed scale efficiency of 27 general hospitals in Greece using DEA analysis. The mean scale efficiency was 94.6%. In contrast, Indian hospitals operate inefficiently [57]. The same conclusion was drawn for Chinese hospitals [58]. Decision makers and administrators in these states should identify the causes of the observed inefficiencies and take appropriate measures to increase the efficiency of these hospitals.

Concerning HMOs, a study found that HMOs with Medicaid patients are significantly less efficient than are other HMOs [59].

Seven articles investigated *Scale Efficiency of Hospitals* [60]. First, Hefty [8] reviewed the progress made in the study of economies of scale in hospitals, finding that the long-term average cost curve appears to be U-shaped, with minimum average costs at the level of 200–300 beds. However, some authors found that economies were exhausted in hospitals with over 10,000 discharges annually [61]. Constant returns to scale also prevailed in Greek public hospitals [62, 63] and in Washington State hospitals in 1988–1993 [64]. Preyra and Pink [65] examined economies of scale in the years preceding restructuring of the hospital sector in the Province of Ontario. Using index and direct approaches, the authors examined a variety of potential reconfigurations and found that there were indeed large-scale unexploited gains achievable from strategic consolidation in the hospital sector.

Concerning *Source of inefficiency*, some authors identify the wasting of resources as a source of inefficiency [16]. However, the hospital’s size was a major source of inefficiency. In addition, bed occupancy ratio appeared to affect both technical and scale efficiency in a rather interesting way [1].

Two articles discussed *Technical and Scale efficiencies’ effect on Quality of care*. The authors found a significant correlation between efficiency score and quality of care [66, 67].

Two articles were collocated under the topic *Effect of market and organizational structure on hospital efficiency*. One of these [68] analysed how the different organizational models adopted in Italy’s healthcare services and patient mobility might affect healthcare efficiency at the regional level. The analysis of the regression results indicated that the national reimbursement system produced a significantly negative effect on healthcare efficiency.

In the second study, Carey [69] discussed about the U.S. multihospital systems, which are organizational structures consisting of two or more hospitals that are separate as physical entities yet share common ownership. In particular, the author estimated a stochastic frontier cost function to test for inefficiency differences among system hospitals having common strategic and/or structural characteristics. Author concluded that system hospitals that centralized around physician arrangements and insurance products display the smallest deviations from the least cost locus. This suggests efficiency benefits from organization of physician and insurance activities at the system level and potential efficiency gains from hospital consolidations. A consolidated firm may be capable of realizing lower costs for a given quantity and quality of services by exploiting economies of scale and scope.

Six articles analysed the *Efficiency effect of health reform*. Authors provided insights into how hospitals responded to the pressure for increased efficiency and quality introduced by the reforms by the adoption of management contracts [70, 71] and by the introduction of the DRG-based payment system [72]. Results showed an improvement in hospital performance primarily driven by quality increases. Specifically, productivity growth was primarily due to technical and scale efficiency changes rather than from technological change.

Aletras et al. [73] estimated the efficiency effect of Greek National Health System reform, which required hospitals to operate as administrative and economic decentralized units under the control of Regional Health Systems. Surprisingly, the analysis indicated that technical and scale efficiencies were reduced following the policy changes. The expected benefits from the reform had not in general been achieved, at least in the short term.

Fidler et al. [74] analysed a decade of experience in Austria and Estonia in restructuring and reorganizing hospital care. In these countries, the incorporation of hospitals and horizontal integration through the creation of holding companies or hospital networks under private law was a viable tool to combine market incentives for management while maintaining public ownership and at the same time achieving efficiency gains and economies of scale. Hospital mergers seemed to stem from a conviction among policy makers that larger hospitals lead to lower average costs and improve clinical outcomes. Instead, employees believed the merger had neither generated economy of scale advantages nor substantial quality improvement; it seemed more rewarding to them to promote cross-functional collaboration together with clinical specialization.

In contrast, Kristensen et al. [75] analysing whether the configuration of Danish public hospitals was subject to economies of scale and scope prior to restructuring plans, found moderate-to-significant economies of scale and scope. This analysis indicated that the Danish hospital sector was characterized by unexploited gains from consolidation.

Finally, 3 articles focussed on *Effect of Ownership on Hospital efficiency*. Some authors found that public hospitals are generally more efficient than are private hospitals [6] and that non-profit hospitals were more efficient than for-profit hospitals [76]. Specifically, for-profit hospitals with between 100 and 249 beds and those with more than 400 beds had lower technical efficiency scores compared with their non-profit peers. Moreover, teaching hospitals were generally more efficient than non-teaching hospitals [77].

### Research topic in a hospital setting

Table 9 shows the frequency distribution of articles published in Health Care Science and Services journals by research topic in a hospital setting. Most articles investigated on these topics using a sample of *General/Acute-care Hospitals* (47%). Sixteen articles employed a *mixed sample*. Specifically, 14 articles analysed technical and scale efficiency using a sample composed of General/Acute-care and Teaching Hospitals. Two articles conducted analyses within a sample composed of district, regional, psychiatric and specialized hospitals [63], and by central, district and level 1 hospitals (level 1 hospitals provide a limited range of specialties and refer patients to other types of hospitals) [71].

**Table 9:** Frequency distribution of articles published in Health Care Science and Services journals by research setting.

RESEARCH SETTING					
**ACCORDING TO SERVICE**	**1969–1989**	**1990–2000**	**2001–2014**	**TOTAL**	**TOTAL%**
General/Acute-care hospitals	1	4	16	21	47%
District hospitals	0	0	2	2	4%
HMOs	0	1	0	1	2%
Hospital units	0	1	2	3	7%
Teaching Hospitals	0	0	0	0	0%
Mixed sample	3	1	12	16	35%
Non-specified	0	0	2	2	4%
**TOTAL**	*4*	*7*	*34*	*45*	*100%*
**ACCORDING TO LOCATION**	**1969–1989**	**1990–2000**	**2001–2014**	**TOTAL**	**TOTAL%**
Urban Hospitals	0	0	0	0	0%
Rural Hospitals	0	0	0	0	0%
All Types	3	3	20	26	58%
Non-Specified	1	4	14	19	42%
**TOTAL**	*4*	*7*	*34*	*45*	*100%*
**ACCORDING TO OWNERSHIP**	**1969–1989**	**1990–2000**	**2001–2014**	**TOTAL**	**TOTAL%**
*Government Hospitals*					
Public Hospitals	1	3	20	24	54%
*Non-Government Hospitals*					
Private Not-For-Profit	0	0	0	0	0%
Private For Profit	0	0	0	0	0%
*All types*	0	0	0	0	0%
*Mixed sample*	3	4	12	19	42%
*Non-specified*	0	0	2	2	4%
**TOTAL**	*4*	*7*	*34*	*45*	*100%*

Two studies used a sample of *district hospitals* [52, 57]. In both studies, district hospitals were operating inefficiently.

Three articles conducted analyses comparing different *hospital units*. Specifically, one study examined the magnitude of economies of scale in14 non-revenue-producing cost centres in hospitals [61]. Findings suggest that there are substantial economies of scale in small hospitals, but economies are exhausted in hospitals with over 10,000 discharges annually. Green [48] examined data from the state of New York and used queueing analysis to estimate bed unavailability in intensive care units and obstetrics units. Finally, Ketabi [55] evaluated the performance of 23 Cardiac Care Units of hospitals in Isfahan, Iran using DEA.

Two papers *non-specified* types of hospitals were included in the analysis in terms of services offered (4%). One study (2%) focussed on the analysis of technical and scale efficiencies for a sample of 28 *HMOs* in Florida [59].

Concerning *location*, most of the articles (58%) included *rural and urban* hospitals in their analyses, and 19 articles did *not specify* the hospital location (42%). Finally, concerning *ownership*, 24 of 44 articles aimed to evaluate the efficiency of only *public hospitals* (54%); however, 19 articles (42%) used a *mixed sample*. Specifically, 14 articles included in their analysis government and non-government hospitals. Cautious conclusions are that public provision might be potentially more efficient than private hospitals in certain settings. Three articles performed analyses using a sample of private for-profit and not-for-profit hospitals [59, 61, 64]. One article estimated the scale and technical efficiency of a sample of hospitals to identified differences between public and private not-for-profit hospitals [72]. Finally, one article [69] used a sample of non-profit, for-profit and government hospitals.

### Research methods

Table 10 shows the frequency distribution of articles published in Health Care Science & Services journals by research methods. Most of the articles (47%) were *empirical studies*. As described previously, the high frequency of these types of studies is not surprising, given the nature of the topic.

**Table 10:** Frequency distribution of articles published in Health Care Science & Services journals by research method.

RESEARCH METHOD	1969–1989	1990–2000	2001–2014	TOTAL	TOTAL%
Empirical study	0	3	18	21	47%
Descriptive study	0	0	0	0	0%
Theoretical study	0	0	0	0	0%
Review	2	0	1	3	6%
Mixed methods	2	4	15	21	47%
**TOTAL**	*4*	*7*	*34*	*45*	*100%*

These papers examined technical and scale efficiencies of hospitals using parametric or non-parametric methods.

In these journals, we found 3 *review* articles. First, a study of Hefty [8] reviewed the progress that was made in the study of economies of scale in hospitals. Results of the empirical studies reviewed in this article showed that the long-term average cost curve appears to be U-shaped, with costs rising slowly as the scale of production goes beyond the optimal point and with the point of minimum average cost most likely occurring between the 200-bed and 300-bed levels. This study constituted the starting point of our systematic search.

Second, Finkler [49] reviewed and reconciled articles concerning the industry's long-term average cost curve, concluding that large hospitals (over 300 beds) might have a greater potential for scale economies. Finally, Hollingsworth [51] reviewed studies on frontier efficiency measurement with parametric and non-parametric methods.

Concerning *mixed methods*, most of the articles were descriptive/empirical studies (10); six articles were theoretical/descriptive studies and four articles were theoretical/descriptive and empirical studies. Authors included in our paper presented a theoretical explanation of concepts, models and question(s) under analysis. Finally, one article was a theoretical/empirical study.

### Primary data analysis techniques

Table 11 shows the frequency distribution of articles published in Health Care Sciences and Services journals by primary data analysis technique.

**Table 11:** Frequency distribution of articles published in Health Care Sciences and Services journals by PDAT.

PDAT					
**QUANTITATIVE**	**1969–1989**	**1990–2000**	**2001–2014**	**TOTAL**	**TOTAL%**
DEA Analysis	0	1	14	15	34%
Stochastic Frontier Analysis	0	0	2	2	4%
Cost Function Model	0	5	6	11	25%
Queueing Analysis	0	0	2	2	4%
Cobb-Douglas Functional Form	0	0	1	1	2%
Mixed methods	0	1	4	5	11%
None	4	0	5	9	20%
**TOTAL**	*4*	*7*	*34*	*45*	*100%*
**QUALITATIVE**	**1969–1989**	**1990–2000**	**2001–2014**	**TOTAL**	**TOTAL%**
Official database	4	7	29	40	89%
Direct contact	0	0	2	2	4%
Mixed sources	0	0	3	3	7%
Non-specified	0	0	0	0	0%
**TOTAL**	*4*	*7*	*34*	*45*	*100%*

PDAT: Primary Data Analysis Techniques.

Concerning *quantitative methods*, most of the articles employed *DEA analysis* to test technical and scale efficiencies in the hospital sector (34%).

Eleven studies used a *Cost function model* to estimate hospital productivity (25%).

Nine articles did not employ quantitative methods (20%).

Specifically, three studies were reviews [8, 49, 51], five were theoretical/descriptive studies [46, 50, 60, 67, 74], and one study was a descriptive study [43].

Concerning *qualitative methods*, over half of the articles (89%) used data collected from *official databases*. Only two articles collected data from *direct contact* with hospitals under analysis.

Specifically, in one study, one author visited the entire population of the hospitals under analysis and reviewed their input and output records [2].

In another study, the author used a questionnaire sent to a hospital to explore responses to the merger of the hospital [46].

Three studies used *mixed data sources*.

In Aletras et al. [73], the authors integrated data, sending electronic and an ordinary mailing of letters to managers of hospitals.

Ketabi [55] evaluated the performance of 23 Cardiac Care Units of hospitals in Isfahan, Iran. Data concerning the productivity of these units were obtained from the monthly archives of the Deputy for Care from Isfahan Medical University. In the next stage, a questionnaire was prepared using the input of measures from preliminary interviews with the hospitals’ authorities.

Finally, Masiye [16] estimated technical and scale efficiencies of a sample of hospitals in Zambia by collecting data from the official database of the Ministry of Health and direct visits to individual’s hospital.

### Studies published in Medicine journals

In this section, we analyse studies on scale efficiency in the hospital sector published by Medicine journal.

### Frequency distribution of articles by Medicine journal

Table 12 shows the frequency distribution of articles published by Medicine journals.

We found 12 articles.

**Table 12:** Frequency distribution of articles published by Medicine journal.

JOURNAL	1969–1989	1990–2000	2001–2014	TOTAL	TOTAL%
*SSM*	0	0	3	3	25%
*ARPH*	0	1	0	1	8%
*BMJ*	0	1	0	1	8%
*BST*	0	0	1	1	8%
*EJPH*	0	0	1	1	8%
*ICM*	0	0	1	1	8%
*IRCMJ*	0	0	1	1	8%
*JAMA*	0	0	1	1	8%
*JCH*	1	0	0	1	8%
*MC*	0	1	0	1	8%
**TOTAL**	*1*	*3*	*8*	*12*	*100%*

SSM: Social Science & Medicine; ARPH: Annual Review of Public Health; BMJ: British Medical Journal; BST: Bioscience Trends; EJPH: European Journal of Public Health; ICM: Intensive Care Medicine; IRCMJ: Iranian Red Crescent Medical Journal; JAMA: The Journal of American Medical Association; JCH: Journal of Community Health; MC: Medical Care.

The highest number of articles (3) was found in the Social Science & Medicine journal (SSM).

### Research topics

Table 13 shows the frequency distribution of articles published in Medicine Journals by research topic.

**Table 13:** Frequency distribution of articles published in Medicine Journal by research topic.

TOPIC	1969–1989	1990–2000	2001–2014	TOTAL	TOTAL%
*Hospital cost efficiency*					
Hospital cost efficiency	0	0	2	2	17%
Hospital mergers and cost saving	1	1	0	2	17%
Optimal size of hospitals	0	1	2	3	25%
*Technical and Scale Efficiencies of Hospitals*					
Frontier Efficiency Measurement	0	0	0	0	0%
Technical and Scale efficiencies score	0	0	1	1	8%
Scale efficiency of hospitals	0	0	1	1	8%
Sources of inefficiency	0	0	0	0	0%
Technical and Scale efficiencies effect on Quality of care	0	0	0	0	0%
Effect of market and organizational structure on hospitals’ efficiency	0	0	0	0	0%
*Effect of healthcare reforms*, *Managerial aspects and ownership on hospital efficiency*					
Efficiency effect of health reforms	0	1	2	3	25%
Effect of ownership on hospital efficiency	0	0	0	0	0%
**TOTAL**	*1*	*3*	*8*	*12*	*100%*

In general, Medicine journals publish research articles, both empirical and theoretical, on health issues to inform current research, policy and practice in all areas of common interest to social scientists, health practitioners, and policy makers. Journals publish material relevant to any aspect of health from a wide range of social science disciplines and relevant material concerning issues of health, health care, clinical practice, health policy and organization.

We found many articles that argue about the implications of mergers and of health reform on hospitals’ efficiency and on quality of care.

Two articles focussed on the evaluation of a *Hospital’s cost efficiency*. Specifically, the authors analysed the cost characteristics of a hospital, concluding that, in general, considering the conclusions concerning economies of scale and economies of scope, larger but more specialized hospitals might be more cost effective [78]. However, the authors found that, in some cases, the reduction of surplus production factors could play a major role in cost reduction of hospitals and health sectors [79].

Concerning *Hospital mergers and cost saving*, evidence seemed to indicate that hospital costs could be reduced through the consolidation of some, or even all, hospital services [80], improving short-term financial performance [81].

Three articles discussed the *optimal size of hospitals*. Most of these studies argued that larger hospitals benefit from the exploitation of economies of scale. However, Posnett [82] discussed the existence of gains in terms of economies of scale in large hospitals, concluding that evidence from research did not support any general presumption that larger hospitals benefit from economies of scale or that service concentration leads to improved outcomes for patients. In contrast, some authors observed that as one hospital's unit increases in size, the cost per patient per day falls [83], concluding that economies of scale and scope depend upon the category of the hospital in addition to the number of beds and volume of output [84].

An important contribution concerning hospital size was made by Tsai and Jha [85]. The authors argued that although high-volume institutions do have better outcomes on average, important caveats in the volume-outcome relationship have implications for how hospital mergers should be evaluated with respect to the delivery of health care; larger is not always better.

The volume-outcome relationship varies widely across conditions and outcomes, with the largest benefits occurring among a small number of technically difficult surgical interventions; volume might simply be a proxy for other processes, such as having systems in place to recognize and effectively manage complications. To improve the delivery of high-quality care, hospitals should instead focus on improving the processes that create better outcomes for patients. High-quality hospitals often have large market share because they are recognized as being good hospitals. Relying on increased volume to create quality might be confusing cause and effect.

Gai et al. [86] used DEA to calculate *technical and scale efficiency scores* of a sample of China’s county hospitals. Geographical disparities in health resource allocation and county hospital productivity were noted. From 1993 to 2005, the number of county hospitals increased, and their inputs (number of medical staff, number of beds, value of fixed capital and hospital expenditures) grew rapidly.

However, the amount of both outpatient and inpatient services declined somewhat, particularly in the middle and the western regions. The overall efficiency at the national level decreased slightly. County hospitals in the eastern region tended to have better overall scale and technical efficiency in comparison to the middle and the western regions.

In conclusion, county hospitals are inefficient due to their enlarged scale and the reduced amount of health care services they provide.

Weaver and Deolalikar [84] analysed *economies of scale* using a sample of 654 public Vietnamese hospitals.

They concluded that economies of scale and scope depend upon the category of the hospital in addition to the number of beds and volume of output. Specifically, the measure of economies of scale was 1.09 for central general and 1.05 for central specialty hospitals, with a mean of 516 and 226 beds, respectively, indicating roughly constant returns to scale. The measure was well below one for both provincial general and specialty hospitals, with a mean of 357 and 192 beds, respectively, indicating large diseconomies of scale.

The measure was 1.16 for district hospitals and 0.89 for other ministry hospitals, indicating modest economies and diseconomies of scale, respectively.

Many articles discussed the *effect of health reforms on a hospital’s efficiency*.

Specifically, authors observed that in the context of health care reform, mergers might offer an expeditious means of locally restructuring health services [87, 88].

Post-merger evidence suggested that mergers might reflect two general strategies: elimination of direct acute competitors or expansion of acute-care networks [89].

### Research topic in a hospital setting

Table 14 shows the frequency distribution of articles published in Medicine journals by research topic in hospital setting.

**Table 14:** Frequency distribution of articles published in Medicine journals by research setting.

RESEARCH SETTING					
**ACCORDING TO SERVICE**	**1969–1989**	**1990–2000**	**2001–2014**	**TOTAL**	**TOTAL%**
General/Acute-care hospitals	1	3	5	9	75%
District hospitals	0	0	0	0	0%
HMOs	0	0	0	0	0%
Hospital units	0	0	1	1	8%
Teaching Hospitals	0	0	0	0	0%
Mixed sample	0	0	2	2	17%
Non-specified	0	0	0	0	0%
**TOTAL**	*1*	*3*	*8*	*12*	*100%*
**ACCORDING TO LOCATION**	**1969–1989**	**1990–2000**	**2001–2014**	**TOTAL**	**TOTAL%**
Urban Hospitals	0	0	0	0	0%
Rural Hospitals	0	0	0	0	0%
All types	0	2	2	4	33%
Non-Specified	1	1	6	8	67%
**TOTAL**	*1*	*3*	*8*	*12*	*100%*
**ACCORDING TO OWNERSHIP**	**1969–1989**	**1990–2000**	**2001–2014**	**TOTAL**	**TOTAL%**
*Government Hospitals*					
Public Hospitals	1	1	6	8	67%
*Non-Government Hospitals*					
Private Not-for-profit	0	0	0	0	0%
Private For Profit	0	0	0	0	0%
*Mixed sample*	0	2	1	3	25%
*All types*	0	0	0	0	0%
*Non-specified*	0	0	1	1	8%
**TOTAL**	*1*	*3*	*8*	*12*	*100%*

Most studies focussed on the analysis of scale efficiency in *General/Acute-care hospitals* (75%).

Most of these articles were published in the period 2001–2014.

Only two articles used a *mixed sample*.

Specifically, Smet [78] analysed cost characteristics of a sample composed of General Acute-care hospitals and teaching hospitals.

Weaver and Deolalikar [84] investigated the performance (economies of scale and scope) of Vietnamese public hospitals using a sample composed of general, specialty, district and ministry hospitals.

The authors found that economies of scale and scope depended upon the category of the hospital in addition to the number of beds and volume of output.

Finally, one study used a sample of Intensive care units in different hospitals to compare efficiency levels [83].

Concerning *hospital location*, most of the articles did *not specify* this aspect (67%).

In contrast, 4 studies included *rural* and *urban hospitals* (33%).

Concerning *hospitals ownership*, over half of the articles included only *public hospitals* (67%); only 3 articles considered *public* and *private hospitals* (25%), and only one article failed to specify hospital ownership (8%).

### Research methods

Table 15 shows the frequency distribution of articles published in Medicine journals by research method. Three studies were *empirical analyses* (25%) [83, 86, 87].

**Table 15:** Frequency distribution of articles published in Medicine journals by research method.

RESEARCH METHOD	1969–1989	1990–2000	2001–2014	TOTAL	TOTAL%
Empirical study	0	0	3	3	25%
Descriptive study	0	0	0	0	0%
Theoretical study	0	1	0	1	8%
Review	0	1	0	1	8%
Mixed methods	1	1	5	7	58%
**TOTAL**	*1*	*3*	*8*	*12*	*100%*

One study was a *theoretical study* [82], and one was a *review* [81].

Concerning *mixed methods*, 3 studies were theoretical/descriptive studies [80, 85, 89], and 3 studies were descriptive/empirical studies [79, 84, 88]. One study was a theoretical/descriptive and empirical study [78].

### The primary data analysis techniques

Table 16 shows the frequency distribution of articles published in Medicine journals by primary data analysis technique.

**Table 16:** Frequency distribution of articles published in Medicine journals by PDAT.

PDAT					
**QUANTITATIVE**	**1969–1989**	**1990–2000**	**2001–2014**	**TOTAL**	**TOTAL%**
DEA Analysis	0	0	3	3	25%
Stochastic Frontier Analysis	0	0	0	0	0%
Cost Function Model	0	0	3	3	25%
Queueing Analysis	0	0	0	0	0%
Cobb-Douglas Functional Form	0	0	0	0	0%
Mixed methods	0	0	1	1	8%
None	1	3	1	5	42%
**TOTAL**	*1*	*3*	*8*	*12*	*100%*
**QUALITATIVE**	**1969–1989**	**1990–2000**	**2001–2014**	**TOTAL**	**TOTAL%**
Official database	1	2	7	10	83%
Direct contact	0	1	1	2	17%
Mixed sources	0	0	0	0	0%
Non-specified	0	0	0	0	0%
**TOTAL**	*1*	*3*	*8*	*12*	*100%*

PDAT: Primary Data Analysis Techniques.

Five articles did not use any quantitative data analysis technique. Specifically, one article was a theoretical/descriptive study [80]; the authors discussed the question of hospital mergers and potential gains. Snail and Robinson [81] presented a review and assessed the state of empirical research on hospital organizational change.

Bogue et al. [89] presented the results of a unique survey on the effects on hospitals after mergers. The authors concluded that in the context of health care reform, mergers might offer an expeditious means to restructure health services locally. Evidence on the post-merger uses of hospitals and concerning the reasons given for mergers suggests that mergers might reflect two general strategies: elimination of direct acute competitors or expansion of acute-care networks.

Posnett [82] discussed the existence of gains in terms of economies of scale in large hospitals and concluded that studies did not support any general presumption that larger hospitals benefit from economies of scale.

Finally, Tsai and Jha [85] was a theoretical/descriptive study.

Concerning qualitative methods, most of the articles (14) used official databases as data sources. Two articles employed data from direct contact with hospitals composing the sample of analysis.

### Studies published in Operations Research and Management Science journals

In this section, we analysed articles published on scale efficiency in the hospital sector by *Operations Research and Management Science* journals.

### Frequency distribution of articles by Operations Research and Management Science journal

Table 17 shows the frequency distribution of 23 articles published by 6 *Operations Research and Management Science journals*.

The most articles (9) were published in Health Care Management Science (HCMS), followed by European Journal of Operational Research (EJOR).

**Table 17:** Frequency distribution of articles published by Operations research & Management Science journals.

JOURNAL	1969–1989	1990–2000	2001–2014	TOTAL	TOTAL %
*EJOR*	1	1	5	7	30%
*HCMS*	0	3	6	9	39%
*ITOR*	0	0	1	1	4%
*JORS*	0	1	2	3	13%
*MS*	2	0	0	2	9%
*PMM*	0	1	0	1	4%
**TOTAL**	*3*	*6*	*14*	*23*	*100%*

EJOR: European Journal of Operational Research; HCMS: Health Care Management Science; ITOR: International Transactions in Operational Research; JORS: Journal of the Operational Research Society; MS: Management Science; PMM: Public Money & Management.

### Research topics

Table 18 shows the frequency distribution of articles published by *Operations Research and Management Science journals* by research topic in the period 1969–2014.

**Table 18:** Frequency distribution of articles published in Operations research & Management journals by research topic.

TOPIC	1969–1989	1990–2000	2001–2014	TOTAL	TOTAL%
*Hospital cost efficiency*					
Hospital cost efficiency	0	3	2	5	22%
Hospital mergers and cost saving	0	0	2	2	9%
Optimal size of hospitals	0	0	0	0	0%
*Technical and Scale Efficiencies of Hospitals*					
Frontier Efficiency Measurement	0	0	0	0	0%
Technical and Scale Efficiencies score	0	1	3	4	17%
Scale efficiency of hospitals	3	1	2	6	26%
Sources of inefficiency	0	0	2	2	9%
Technical and Scale Efficiencies effect on Quality of care	0	0	0	0	0%
Effect of market and organizational structure on hospitals’ efficiency	0	0	1	1	4%
*Effect of healthcare reforms*, *Managerial aspects and ownership on hospital efficiency*					
Efficiency effect of health reforms	0	1	1	2	9%
Effect of ownership on hospital efficiency	0	0	1	1	4%
**TOTAL**	*3*	*6*	*14*	*23*	*1*

Five articles focussed on *Hospital cost efficiency*.

Some authors, assessing the efficiency of a sample of hospitals and focussing on cost efficiency and production, found that hospital size does not seem to play a differentiating role [90].

In contrast, other authors [91, 92] who explored the efficiency of hospitals observed that larger hospitals displayed higher cost efficiency, higher allocative efficiency and higher technical efficiency than did their smaller counterparts. Additionally, significant economies of scale were found in emergency department care [93], supporting a possible expansion of emergency department size policy to improve the cost efficiency of these services.

Another factor used as a strategy to increase efficiency levels and reduce hospitals costs was the diversification of the output-mix offered [94].

The topic *Hospitals’ mergers and cost saving* was discussed in only two articles.

The authors showed potential gains from improving technical efficiency and the exploitation of economies of scope from mergers [5, 95].

*Technical and scale efficiency scores* of hospitals were analysed in 4 articles.

Generally, also in this context, authors observed that mergers increased a hospital's efficiency level [96, 97, 98, 99].

Most articles in this category of journals focussed on methods analysis and on calculation of *Scale efficiency* in hospitals.

Authors discussed returns to scale in hospitals [100] and the most productive scale size, using the DEA model proposed by Charnes et al. [101].

In this context, Banker et al. [102] stated, "for most applications, it is more meaningful to work with the mpss concept". Zhu and Shen [103] also studied the issue of using Banker's most productive scale size concept to characterize DMUs' returns to scale. Their paper laid out the precise condition under which the MPSS concept fails to work. That is, linearly dependent relationships in a set of efficient DMUs might cause the MPSS concept not to work.

Concerning hospitals’ efficiency measured in terms of beds, in some cases, authors observed that increasing returns to scale could be exploited up to a capacity of approximately 200 beds [104].

In other cases, the results of analysis showed increasing returns to scale among hospitals above the median size (more than 300 beds) [105].

Two articles discussed potential *Sources of inefficiency* in the hospital sector.

Specifically, teaching status (management variable) was found to have significant effects on inefficiency of general service costs [106]; on average, teaching hospitals were less efficient (in term of converting general services to patient-day outputs). Concerning sources of inefficiency, Peyrache [107] also contributed in this field, showing that a certain percentage of inefficiency for Australian hospitals was attributable to size inefficiencies.

The *Effect of market and organizational structure on hospitals’ efficiency* was analysed by Ancarani et al. [108]. The authors investigated the effect of managerial and organizational aspects on Italian hospital wards’ efficiency. Results showed that both decisions internal to the ward and exogenous re-organizations affect the ward’s efficiency and suggested that these variables were more significant in explaining efficiency than were environmental ones.

The *Efficiency effect of health reforms* was a topic discussed in two articles. Sommersguter [109] explored productivity changes in Austrian hospitals after and before hospital financing reform. The results illustrated a considerably positive shift in technology between 1996 and 1998, whereas the intended enhancement in technical efficiency had not yet occurred. Ferrier et al. [110] discussed the efficacy of certificate of need (CON) regulations using US hospitals’ data. Results showed that the CON regulation seemed to improve the mix allocation among hospitals; conversely, the regulation also seemed to constrain the size of hospitals, which have difficulty achieving the most productive scale size. Finally, Gruca and Nath [111] investigated the *effect of ownership*, *size*, *and location on the relative technical efficiency* of community hospitals in Ontario, which has a single payer system. Concerning ownership, in contrast to US-based research, the authors found no significant differences in efficiency across ownership types (government, religious or secular non-profit).

The authors highlighted this finding as key because prior research using data from the United States suffered from two potential limitations. Pooling all hospitals in a single model could result in a bias against certain types of hospitals. In addition, none of the previous studies considered the importance of payer mix in their models.

### Research topic in a hospital setting

Table 19 shows the frequency distribution of articles published in Operations research & Management journals by research topic in a hospital setting. The popular choice of settings for scale efficiency studies was *mixed sample* (35%), with 8 studies. One study [94] focused on potential diversification economies as a strategy to increase efficiency levels, using a sample composed of all types of diversified and specialized hospitals. The results showed that the majority of hospitals could increase their efficiency and reduce their costs by diversification of the offered output mix. One study [109] used a sample of government and non-government hospitals to explore productivity changes after and before hospital financing reform. Four studies [5, 95, 106, 107] analysed hospital productivity of a sample of general acute-care hospitals, including teaching hospitals. In 2008, one study analysed the technical efficiency of a sample of 88 hospitals including general, specialized, district and community, and teaching hospitals [98]. The main findings were that the three largest hospitals were performing well; general hospitals were the most inefficient; community hospitals were the most efficient; and specialized hospitals were in an intermediate group. One study [88] investigated the production technology of Portuguese hospitals and estimated their efficiency. The sample was composed of single hospitals (SH), a single hospital unit, hospital centres (HC), or local health units (LHU). The technologies of the Portuguese hospitals demonstrate overall non-increasing returns to scale. Hospitals should reach an optimal scale by reducing their cost levels and increasing their assets. Seven studies performed analysis including only *general/acute-care hospitals* (30%). Five studies did *not specify* the hospital type included in the sample according to service offered (22%). Three studies (13%) performed analyses comparing different *hospital units*. Results showed that both decisions internal to the ward and exogenous re-organizations affect the ward’s efficiency, and the results also suggested that these variables are more significant in explaining efficiency than are environmental ones [93, 103, 108]. However, one of these studies analysed economies of scale in emergency departments [92], supporting a possible expansion of ED size policy to improve the cost efficiency of ED services. Concerning *hospital location*, most of these studies did not specify this aspect (52%). Nine studies (39%) included *all types* of hospitals (rural and urban). Empirical results confirmed that urban hospitals, despite the high demand for services, are very much better supplied with resources than are corresponding rural hospitals. The concentration of health services in city centres does have negative implications for efficiency [90]. Two studies considered only *urban hospitals* (9%). Concerning *hospital ownership*, 7 studies included only *public hospitals* in our sample (30%), followed by studies that did not specify this aspect (30%). Concerning the *mixed sample* (21%), four studies considered public and private hospitals, and one study used a sample composed of private for profit and not-for-profit hospitals [96]. Finally, four studies included *public*, *private and church hospitals* (17%).

**Table 19:** Frequency distribution of articles published in Operations research & Management journals by research setting.

RESEARCH SETTING					
**ACCORDING TO SERVICE**	**1969–1989**	**1990–2000**	**2001–2014**	**TOTAL**	**TOTAL%**
General/Acute-care hospitals	2	1	4	7	30%
District hospitals	0	0	0	0	0%
HMOs	0	0	0	0	0%
Hospital units	0	1	2	3	13%
Teaching Hospitals	0	0	0	0	0%
Mixed sample	0	2	6	8	35%
Non-specified	1	2	2	5	22%
**TOTAL**	*3*	*6*	*14*	*23*	*100%*
**ACCORDING TO LOCATION**	**1969–1989**	**1990–2000**	**2001–2014**	**TOTAL**	**TOTAL%**
Urban Hospitals	0	0	0	0	0%
Rural Hospitals	0	0	2	2	9%
All types	1	2	6	9	39%
Non-Specified	2	4	6	12	52%
**TOTAL**	*3*	*6*	*14*	*23*	*100%*
**ACCORDING TO OWNERSHIP**	**1969–1989**	**1990–2000**	**2001–2014**	**TOTAL**	**TOTAL%**
*Government Hospitals*					
Public Hospitals	1	1	5	7	30%
*Non-Government Hospitals*					
Private Not-for-profit	0	0	0	0	0%
Private For Profit	0	0	0	0	0%
*All types*	1	0	3	4	17%
*Mixed sample*	0	3	2	5	21%
*Non-specified*	1	2	4	7	30%
**TOTAL**	*3*	*6*	*14*	*23*	*100%*

### The research method

Table 20 shows the frequency distribution of articles published in Operations research & Management journals by research method. Data presented revealed that *empirical study* methods were the most frequently used, with 11 articles (48%). Only one study was a *descriptive study* (4%) [103].

**Table 20:** Frequency distribution of articles published in Operations research & Management journals by research method.

RESEARCH METHOD	1969–1989	1990–2000	2001–2014	TOTAL	TOTAL%
Empirical study	2	3	6	11	48%
Descriptive study	0	1	0	1	4%
Theoretical study	0	0	0	0	0%
Review	0	0	0	0	0%
Mixed methods	1	2	8	11	48%
**TOTAL**	*3*	*6*	*14*	*23*	*100%*

Concerning the *mixed methods*, 8 articles were descriptive/empirical studies. Seven of these articles were published in the third period (2001–2014). Two studies were theoretical/empirical, and one was a theoretical/descriptive.

### Primary data analysis techniques

Table 21 shows the frequency distribution of articles published in Operations research & Management journals by primary data analysis technique. Concerning *quantitative methods*, *DEA analysis* was preferred (61%).

**Table 21:** Frequency distribution of articles published by Operations research & Management journals by PDAT.

PDAT					
**QUANTITATIVE**	**1969–1989**	**1990–2000**	**2001–2014**	**TOTAL**	**TOTAL%**
DEA analysis	2	5	7	14	61%
Stochastic Frontier Analysis	0	0	0	0	0%
Cost Function Model	0	0	1	1	4%
Queueing Analysis	0	0	0	0	0%
Cobb-Douglas Functional Form	0	0	0	0	0%
Mixed methods	1	1	6	8	35%
None	0	0	0	0	0%
**TOTAL**	*3*	*6*	*14*	*23*	*100%*
**QUALITATIVE**	**1969–1989**	**1990–2000**	**2001–2014**	**TOTAL**	**TOTAL%**
Official database	2	6	11	19	83%
Direct contact	0	0	0	0	0%
Mixed sources	0	0	3	3	13%
Non-specified	1	0	0	1	4%
**TOTAL**	*3*	*6*	*14*	*23*	*100%*

PDAT: Primary Data Analysis Techniques.

Eight studies employed *mixed methods* (35%). First, Banker et al. [102] presented a comparative application of DEA analysis and the Translog cost function to analyse hospital production.

Two studies [95,109] applied DEA analysis to explore technical and scale efficiencies of a sample of hospitals and the Malmquist index to analyse productivity changes after and before hospital reform. Blank and Eggink [97] applied the shadow cost function model and SFA to assess technical and allocative efficiency of the Dutch general hospital industry.

Two studies applied DEA analysis and a Tobit model to analyse the determinants of the hospital’s overall inefficiency and its respective input inefficiencies [106] and to analyse the effect of managerial and organizational aspects on hospital wards’ efficiency [108].

Oliveira and Bevan [98] used a hierarchical fixed effects model and a multi-level random intercepts and slopes model to investigate technical efficiency for a sample of hospitals.

Finally, Peyrache [107] employed DEA analysis and the directional distance function to investigate hospital mergers and potential gains.

Only one study used the *cost function model* to analyse scale efficiency for a sample of hospital units [93].

Concerning *qualitative methods*, most articles used *official records* (83%). Three articles collected data using *mixed sources* (13%).

Specifically, two studies [92,98] used official databases and direct contact with hospitals (missing elements were directly requested of the hospitals); in one article, data were collected from official records (official web Ministry of health) and face-to-face interviews based on a questionnaire [108].

One article did *not specify* data sources.

## Discussion and limitations

The aim of this article has been to explore the status of research on scale efficiency in the hospital sector to identify gaps and suggest ideas for future research.

For an initial discussion about the survey results it could be useful to try connecting academic fields. In particular, in order to give policy indication regarding hospital size could be useful connecting results of the studies from the different academic fields.

The majority of the studies published in Business and Economics journals were focused on the question of hospital mergers and related cost saving and efficiency. Authors showed that hospitals’ cost inefficiency was often due to using too many input resources (number of personnel and beds–technical inefficiency); also the use of a wrong mix of resources (allocative inefficiency) also raised costs. In terms of beds, studies showed increasing return to scale among hospitals with 300 beds and until 600 beds. Authors concluded that hospitals could substantially reduce costs by adjusting their level and mix of input usage, thus reducing costs without sacrificing access.

Studies published in Health Care Science and Services journals highlighted that the effects and the benefits of the mergers depend on the type of the organizations involved; in particular the aggregations among similar organizations appears to be very effective. They also confirmed that the economies of scale are greater when the new entity has a number of beds between 250 and 300, and with less than 10,000 annual discharges.

From studies published in Medicine journals it was possible to draw interesting conclusions about the relationship between volumes and outcomes. In particular, authors concluded that there is no evidence that the increase in size may lead to outcome improvements.

As regard studies published in Operation research and Management science journals some authors assessed the efficiency of a sample of hospitals, focusing on cost efficiency and production.

Most of the articles concluded that the efficiency is influenced both by the size of organizations that from the offered output mix. They confirmed what was said in the previous fields: economies of scale are evident for hospitals with 200–300 beds. Diseconomies of scale can be expected to occur below 200 beds and above 600 beds.

In conclusion, our systematic search started with 4 search questions:

1. Have mergers contributed to enhance hospitals efficiency?
2. Which is the optimal size of hospitals in terms of beds?
3. Which factors influenced the hospitals scale efficiency?
4. Finally, which are the most methods used in literature to analyse the hospitals scale efficiency?

According to this literature review it is possible to answer these questions in the following way:

1. Studies analysed in this review showed that economies of scale are present for merging hospitals. Results supported the current policy of expanding larger hospitals and restructuring/closing smaller hospitals [27].
2. In terms of beds, studies reported consistent evidence of economies of scale for hospitals with 200–300 beds [8]. Diseconomies of scale can be expected to occur below 200 beds and above 600 beds [6].
3. Many factors influence scale efficiency level [112, 113, 114]. Economies of scale depended upon the category of the hospital in addition to the number of beds and volume of output [84, 115, 116, 117]. Therefore, many studies used a sample composed of different types of hospitals in terms of services offered, ownership and location. According to services offered, because of their distinct natures and unique production processes, hospitals including long-term care (the latter being defined as hospitals having average length of stay above 25 days), or specialty hospitals, such as psychiatric, children’s and cancer centres, were excluded from the samples. However, these hospitals have different cost structures and generally do not provide emergency services. In contrast, the popular choice of setting for scale efficiency studies was General/Acute-care hospitals. Additionally, some studies included teaching hospitals in their samples. Teaching activities are an important cost-driving factor, and hospitals that have a broader range of specialization are relatively more costly. According to ownership, public hospitals are more efficiently than other types. Finally, according to location, urban hospitals used resources more efficiently.
4. Concerning methods, many works on scale efficiency were empirical studies, given the nature of this topic. Authors calculated the technical and scale efficiencies of sample hospitals and provided several contributions for research and practice to understand factors affecting hospitals’ efficiency. The techniques used are primarily based on non-parametric DEA, but there is an increasing use of parametric techniques such as SFA, as reported in Table 22, which shows the most 10 cited articles founded in our review. The 10 most cited articles were identified using the Google Scholar and Science Citation Index database of the Institute for Scientific Information. Data were extracted by one author. The most cited papers identified seminal contributions and originators of landmark methodological aspects of scale efficiency in hospital sector, representing a good guide for the measurement and productivity of health care services. A small number of reviews were identified concerning scale efficiency in the hospital sector. Accordingly, the aim of our systematic search is to provide several contributions for both research and practice.

**Table 22:** Top 10 articles list

Authors	Year	Title	Main Conclusions	RANK	No of citations
Banker RD	1984	Estimating most productive scale size using data envelopment analysis	Application of DEA model to a sample of hospitals showed that economies of scale are evident for hospitals with 200 beds.	**1**	1019
Banker RD, Conrad RF, Strauss RP	1986	A comparative application of Data Envelopment Analysis and Translog Methods: an illustrative study of hospital production	Application of translog and DEA models to a sample of North Carolina hospitals revealed that constant returns to scale were present in the hospital industry. The mean mpss for the 29 hospitals was between 110 and 160 beds.	**2**	744
Hollingsworth Bruce	2008	The Measurement Of Efficiency And Productivity Of Health Care Delivery	A review of 317 published papers on frontier efficiency measurement revealed that the techniques used are mainly based on nonparametric data envelopment analysis. There is increasing use of parametric techniques, such as stochastic frontier analysis.	**3**	588
Vita MG	1990	Exploring hospital production relationships with flexible functional forms	The paper estimated a multiproduct variable cost function using data on a sample of California hospitals. The paper's results do not provided strong evidence of either ray scale economies or of weak cost complementarities.	**4**	319
Gaynor, Wilson	1999	Change, consolidation, and competition in health care markets	Authors discussed the potential implications of the restructuring of the health care industry for competition, efficiency, and public policy. Given the increasing reliance on markets to allocate health care resources, health care policy should seek to ensure that these markets work efficiently. Cautious enforcement of the antitrust laws is essential both to prevent monopoly power and to ensure that antitrust enforcement activity does not discourage the growth of new and efficient forms of health care organization.	**5**	317
Linna M.	1998	Measuring hospital cost efficiency with panel data models	This paper investigated the development of hospital cost efficiency and productivity in Finland. Parametric and non-parametric panel models were used to investigated about cost efficiency and productivity of hospitals. The results revealed a 3–5% annual average increase in productivity, half of which was due to improvement in cost efficiency and half due to technological change.	**6**	255
Ferrier GD, Valdmanis V	1996	Rural hospital performance and its correlates	The cost, technical, allocative and scale efficiencies of a sample of rural U.S. hospitals are calculated via linear programming models. A large amount of dispersion in operating efficiency was found within our data set; the majority of the dispersion was due to technical inefficiency. In general, for-profit hospitals were found to outperform not-for-profit and public hospitals. Demand characteristics, quality of care, and the mix of services offered were also found to influence performance.	**7**	235
Zhu, Shen	1995	A discussion of testing DMUs' returns to scale	This paper has laid out the precise condition under which the mpss concept fails to work. That is, linearly dependent relationships in a set of efficient DMUs may cause the mpss concept not to work. As a result, there is an incorrect statement in Chang and Ghu (1991) that attributes a linear production function to the CCR model. It has also been pointed out that the linear dependency condition corresponds to the non unique optimal lambda solution situation in Banker and Thrall (1992). A remedy has been provided to make the mpss concept work under linear dependency (i.e., multiple optimal lambda values).	**8**	217
Green Linda V, Nguyen V.	2001	Strategies for Cutting Hospital Beds: The Impact on Patient Service	This paper developed insights on the impact of size, average length of stay, variability, and organization of clinical services on the relationship between occupancy rates and delays for beds. Data from Beth Israel Deaconess on discharges and length of stay were analyzed. Using target occupancy levels as the primary determinant of bed capacity is inadequate and may lead to excessive delays for beds. Also, attempts to reduce hospital beds by consolidation of different clinical services into single nursing units may be counterproductive. More sophisticated methodologies are needed to support decisions that involve bed capacity and organization in order to understand the impact on patient service.	**9**	194
Vitaliano, DF	1987	On the estimation of Hospital cost-functions	Data from 166 general hospitals in New York State (1981) was used to estimate a quadratic and logarithmic long-run cost function. The author confirm the commonly-held view of a shallow U-shaped average cost curve, concluding that scale economics exist in the hospital sample.	**10**	183

Whilst this systematic search aimed to be rigorous, there are a few limitations. A first limitation of our study is that our results might be affected by publication bias because our analysis is necessarily limited to publicly available papers. In addition, only papers published in English language were reviewed which means that findings from data published in other languages were automatically excluded from the review.

The number of studies that seek to measure health-service efficiency and productivity continues to increase steadily. Research in this area should be reviewed carefully, and the results of studies interpreted and should be used cautiously because the area remains under development.

Our review will provide future scale-efficiency researchers with direction leading to a “new” knowledge base for the scale-efficiency research field.

## Supporting information

S1 Table PRISMA 2009 Checklist. (DOCX)

S1 Appendix Journal List. (DOCX)
