# Supplementary material for: Efficiency and optimal size of hospitals: Results of a systematic search
Source: PLoS One. 2017 Mar 29;12(3):e0174533. doi: 10.1371/journal.pone.0174533 (PMC5371367; doi:10.1371/journal.pone.0174533)
Supplement: S1 Appendix — (DOCX) [file pone.0174533.s002.docx]

# Efficiency and Optimal Size of Hospital: Results of a SystematicSearch

**Supporting Information**

**S2 Appendix**

**Business and Economics Journals founded for scale efficiency in hospital sector**

| Journal Name | Journal Acronyms | Impact Factor 2013 | Journal's Country |
| --- | --- | --- | --- |
| Applied Economics | AEJ | 0.518 | England |
| China Economic Review | CER | 1.142 | USA |
| Economic Inquiry | EI | 1.028 | USA |
| EkonomickyCasopis | EK | 1.142 | Slovakia |
| Economic Modelling | EM | 0.736 | Netherlands |
| Journal of Applied Econometrics | JAE | 1.562 | United Kingdom |
| Journal of Economic Perspectives | JEP | 4.230 | USA |
| Journal of Productivity Analysis | JPA | 0.787 | Netherlands |
| Review of Industrial Organization | RIO | 0.468 | Netherlands |
| Regional Science and Urban Economics | RSUE | 0.971 | Netherlands |
| The Review of Economics and Statistic | RvE&S | 2.718 | Cambridge |
| South African Journal of Economics | SAJEMS | 0.183 | England |
| Economic and Social Review | ESR | 0.200 | Ireland |

**Health Care Sciences and Services journals founded for scale efficiency in hospital sector**

| Journal Name | Journal Acronyms | Impact Factor 2013 | Journal's Country |
| --- | --- | --- | --- |
| BMC Health Services Research | BMC HSR | 1.659 | England |
| European Journal of Health Economics | EJHE | 1.913 | USA |
| Global Health Action | GHA | 1.646 | Sweden |
| Health Care Management Review | HCMR | 1.642 | USA |
| Health Economics | HE | 2.137 | England |
| Health Economics Policy And Law | HEPL | 1.593 | USA |
| Health Policy | HP | 1.725 | Ireland |
| The International Journal Of Health Planning and Management | HPMJ | 0.971 | USA |
| Health Services Research | HSR | 2.491 | USA |
| INQUIRY: The Journal of Health Care Organization, Provision, and Financing | INQ | 0.564 | USA |
| Journal of Health Care for the Poor and Underserved | JHCPU | 0.902 | USA |
| Journal of Health Economics | JHE | 2.254 | Netherlands |
| Journal of Medical Systems | JMS | 1.372 | USA |
| Journal of Rural Health | JRH | 1.771 | USA |

**Medicine journals founded for scale efficiency in hospital sector**

| Journal name | Journal acronyms | Impact Factor 2013 | Journal's Country |
| --- | --- | --- | --- |
| Annual Review of Public Health | ARPH | 6.627 | USA |
| British Medical Journal | BMJ | 16.37 | United Kingdom |
| Bioscence Trends | BST | 1.213 | Japan |
| European Journal of public health | EJPH | 2.728 | United Kingdom |
| Intensive Care Medicine | ICM | 5.544 | USA |
| Iranian Red Crescent Medical Journal | IRCMJ | 0.504 | Netherlands |
| The Journal of American Medical Association | JAMA | 30.387 | USA |
| Journal of community health | JCH | 1.573 | Netherlands |
| Medical Care | MC | 2.941 | USA |
| Social Science & Medicine | SSM | 2.558 | England |

**Operations Research & Management Science journals founded for scale efficiency in hospital sector**

| Journal Name | Journal Acronyms | Impact Factor 2013 | Journal's Country |
| --- | --- | --- | --- |
| European Journal of Operational Research | EJOR | 1.843 | Netherlands |
| Health Care Management Science | HCMS | 0.071 | Netherlands |
| International Transactions in Operational Research | ITOR | 0.481 | Denmark |
| Journal of the Operational Research Society | JORS | 0.911 | United Kingdom |
| Management Science | MS | 1.733 | USA |
| Public Money & Management | PMM | 0.636 | England |
